# Profiling of MicroRNAs Involved in Mepiquat Chloride-Mediated Inhibition of Internode Elongation in Cotton (*Gossypium hirsutum* L.) Seedlings

**DOI:** 10.3389/fpls.2021.643213

**Published:** 2021-02-24

**Authors:** Li Wang, Ying Yin, Xiuxiu Jing, Menglei Wang, Miao Zhao, Juanjuan Yu, Zongbo Qiu, Yong-Fang Li

**Affiliations:** ^1^College of Life Sciences, Henan Normal University, Xinxiang, China; ^2^Henan International Joint Laboratory of Agricultural Microbial Ecology and Technology, Henan Normal University, Xinxiang, China

**Keywords:** mepiquat chloride, internode, microRNA, cotton, target gene

## Abstract

Mepiquat chloride (MC) is the most important plant growth retardant that is widely used in cotton (*Gossypium hirsutum* L.) production to suppress excessive vegetative growth and improve plant architecture. MicroRNAs (miRNAs) are important gene expression regulators that control plant growth and development. However, miRNA-mediated post-transcriptional regulation in MC-induced growth inhibition remains unclear. In this study, the dynamic expression profiles of miRNAs responsive to MC in cotton internodes were investigated. A total of 508 known miRNAs belonging to 197 families and five novel miRNAs were identified. Among them, 104 miRNAs were differentially expressed at 48, 72, or 96 h post MC treatment compared with the control (0 h); majority of them were highly conserved miRNAs. The number of differentially expressed miRNAs increased with time after treatment. The expression of 14 known miRNAs was continuously suppressed, whereas 12 known miRNAs and one novel miRNA were continuously induced by MC. The expression patterns of the nine differentially expressed miRNAs were verified using qRT-PCR. The targets of the known and novel miRNAs were predicted. Four conserved and six novel targets were validated using the RLM-5′ RACE assay. This study revealed that miRNAs play crucial regulatory roles in the MC-induced inhibition of internode elongation. It can improve our understanding of post-transcriptional gene regulation in MC-mediated growth inhibition and could potentially facilitate the breeding of dwarf cotton.

## Introduction

Cotton (*Gossypium hirsutum* L.) is a worldwide cultivated economic crop that provides natural fiber materials and edible oil. One of the major challenges in cotton production is the control of excessive vegetative growth during the entire developmental stage owing to its indeterminate growth habit. Serious production problems, such as auto-shading, fruit abscission, delayed maturity, and reduced yield, often occur under excessive vegetative growth (Zhao and Oosterhuis, 2000). Therefore, the vegetative growth of cotton should be controlled. Mepiquat chloride (MC) is the most commonly used growth regulator in cotton production; it shortens internodes, decreases plant height, and reduces leaf area (Reddy et al., 1990; Rademacher, 2000; Siebert and Stewart, 2006; Curaba et al., 2013; Ren et al., 2013). The compact plant architecture and open canopy by MC application are important for maximizing cotton yield (Gwathmey and Clement, 2010; Ren et al., 2013).

Mepiquat chloride is a well-known gibberellin (GA) biosynthesis inhibitor. Early studies hypothesized that MC specifically inhibits the activity of copalyl diphosphate synthase in the early steps of GA biosynthesis (Shechter and West, 1969; Rademacher, 2000). Recently, we found that MC repressed cell division and elongation of cotton internodes by reducing not only endogenous GA but also auxin and brassinosteroid (BR) contents (Wang L. et al., 2020). Transcriptome profiling showed that MC remarkably reduced the expression of genes related to GA, auxin, BR, and ethylene metabolism and signaling but increased the expression of genes related to cytokinin and abscisic acid (Wang L. et al., 2020). Furthermore, many transcription factors (TFs), including growth regulating factor (GRF), TEOSINTE BRANCHED1/CYCLOIDEA/PCF (TCP), no apical meristem (NAC), myb domain protein (MYB), and squamosa promoter binding protein (SPLs/SBP), which play key modulating functions in plant growth, were significantly altered by MC (Wang L. et al., 2020).

MicroRNAs (miRNAs) are a class of non-coding RNAs with lengths of ∼20–24 nt. miRNAs modulate the expression of their targets post-transcriptionally through mRNA cleavage or translation inhibition in a sequence-specific manner (Addoquaye et al., 2009). Most targets of the conserved miRNAs are TFs or phytohormone-related genes (Rodriguez et al., 2016; Tang et al., 2018), therefore, miRNAs play key roles in many biological processes such as organ development, hormone signal transduction, growth phase switching, and stress response (Gao et al., 2016; Rodriguez et al., 2016; Tang et al., 2018; Yuan et al., 2019). Several miRNAs have been shown to be associated with plant height by modulating internode elongation (Schwab et al., 2005; Chuck et al., 2007; Alonso-Peral et al., 2010; Fu et al., 2012; Gao et al., 2015, 2016; Liu et al., 2017; Zhao et al., 2017; Dai et al., 2018; Jiang et al., 2018; Sun et al., 2019; Yuan et al., 2019). In rice, overexpression of miR319, miR396d, miR164b, and miR535 or suppression of miR159 shortened internodes and caused plant dwarfism (Liu et al., 2017; Zhao et al., 2017; Jiang et al., 2018; Tang et al., 2018; Sun et al., 2019). Transgenic creeping bentgrass (*Agrostis stolonifera*) overexpressing osa-miR396c exhibited shortened internodes (Yuan et al., 2019). Enhanced expression of miR156 reduced plant height in Arabidopsis, rice, maize, alfalfa, and switchgrass (Schwab et al., 2005; Chuck et al., 2007; Fu et al., 2012; Gao et al., 2016; Dai et al., 2018). TCPs regulated by miR319 have been shown to control cell proliferation in plants (Rodriguez et al., 2016). miR396 has been proven to be closely related to cell proliferation and elongation by targeting GRFs (Rodriguez et al., 2016; Tang et al., 2018), and miR396 is associated with the pathways of several hormones such as GA, auxin, and BR (Gao et al., 2015; Tang et al., 2018). miR159 positively regulates organ growth by promoting cell division via the targeting of GAMYBs (Alonso-Peral et al., 2010; Zhao et al., 2017). Suppression of miR159 in rice impaired cell cycle and hormone homeostasis (Zhao et al., 2017).

Although miRNAs associated with plant height have been identified in several crops, limited information is available on miRNAs related to cotton internode elongation. Most studies on cotton miRNAs have focused on miRNA profiling and identification of miRNAs associated with fiber initiation and elongation (Zhao et al., 2019), anther development (Zhang et al., 2018), male sterility (Nie et al., 2018), somatic embryogenesis (Yang et al., 2013), stress response (Xie et al., 2015a), and disease resistance (Shweta et al., 2018). By comparing the miRNA profiles between wild type and dwarf mutants of cotton, An et al. (2015) identified 104 differentially expressed miRNAs (DEG miRNAs) in stem apexes and revealed the roles of miRNAs in controlling cotton plant height. In our previous study, we found that many genes, including TFs, were altered by MC application (Wang L. et al., 2020); thus, we hypothesize that miRNAs may contribute to MC-induced internode elongation inhibition at post-transcriptional level. To identify the miRNAs responsive to MC treatment and reveal their potential roles in MC-mediated growth inhibition, we sequenced four small RNA (sRNA) libraries from cotton internodes at the three-leaf stage at different time points (0, 48, 72, and 96 h) post MC treatment. This study reveals the regulatory roles of miRNAs in cotton internode elongation and provides novel insights into the molecular mechanisms of MC-mediated growth inhibition, which may facilitate the breeding of dwarf cotton.

## Materials and Methods

### Plant Growth and Treatments

Upland cotton (*G. hirsutum* ‘CCRI49’) seeds were obtained from the Institute of Cotton Research of the Chinese Academy of Agricultural Sciences (Anyang, China). Cotton seeds were immersed in water for 8 h at 37°C and then germinated in sand at 28°C in the dark for 3 days. Subsequently, uniform seedlings were transferred to plastic pots filled with aerated half-strength Hoagland solution and grown hydroponically in a growth chamber with a 14-h photoperiod at a 28/20°C day/night temperature cycle and a light intensity of 550 μmol m^–2^ s^–1^.

Based on our previous study (Wang L. et al., 2020), 80 mg/L of MC (Hebei Guoxin ahadzi Biological Technology Co., Ltd., Hejian, Hebei, China) was applied to the cotton seedlings at the three-leaf stage by foliar spraying until the leaf was evenly wetted and started dripping (approximately 4 mL/plant). The upper halves of the second internode were harvested at 0 h (control), 48, 72, and 96 h post MC treatment (hpt), and samples at each time point were collected from 10 seedlings, immediately frozen in liquid nitrogen, and then stored at –80°C for RNA extraction, with three biological replicates for each time point. Morphological phenotypes of control and MC-treated seedlings were observed at 10 days post treatment.

### sRNA Library Construction and Sequencing

Total RNA was extracted from internodes using TRIzol reagent (Invitrogen, Carlsbad, CA, United States) according to the manufacturer’s instructions. The RNA quality and integrity of each sample were evaluated using 1% agarose gel electrophoresis and a Bioanalyzer (Agilent 2100). Equal amounts of total RNA from three biological replicates were mixed and sent to BGI (Shenzhen, China) for the construction of sRNA libraries. sRNAs of 18–30 nt in length were enriched using 15% denatured polyacrylamide gel electrophoresis (PAGE) and ligated to the 3′ and 5′ RNA adaptors. A reverse transcription reaction was then performed to generate cDNA, which was then used for subsequent PCR enrichment. The final PCR products were purified using PAGE (Wang et al., 2015). The constructed libraries were sequenced using the BGISEQ-500RS sequencing platform (BGI, Shenzhen, China).

### Analysis of sRNA Sequencing Data

Raw sequencing reads were first processed by filtering low-quality reads and trimming the adaptor sequences, and eventually, high-quality clean reads (18–30 nt) were acquired. Any sRNAs mapping to rRNAs, tRNAs, snRNAs, protein-coding genes, or repeat sequences were filtered by alignment against the databases of Rfam, Repbase, GtRNAdb, and Silva. Subsequently, the clean reads were aligned to the *G. hirsutum* TM-1 genome (Zhang et al., 2015) using Bowtie tools with no mismatch and multiple mappings allowed. Only the mapped sRNAs were subjected to identification of cotton-known miRNAs. The mature miRNA sequences from all plant species were downloaded from miRBase 22.1^1^ and combined to obtain all known plant miRNA sequences. Cotton known miRNAs in internodes were identified by BLASTN search against all known plant miRNA sequences. Only sRNAs with not more than two mismatches were identified as cotton-known miRNAs. The remaining sRNAs were used to predict novel cotton miRNAs using the miRdeep2 software (Friedländer et al., 2012).

The flanking genome sequences (150 nt upstream and 150 nt downstream) of sRNAs were used to predict the secondary hairpin structures using RNAfold software.^2^ The secondary structures of the novel miRNAs were further checked manually using the MFOLD (Zuker, 2003). The criteria we used to identify novel miRNAs were based on a recent article (Axtell and Meyers, 2018).

### Identification and Validation of Differentially Expressed miRNAs

For miRNA expression analysis, tags per million (TPM) were utilized to normalize the miRNA expression levels as follows: TPM = (read count/mapped reads)^∗^1,000,000. Differential expression analysis of miRNAs between the two samples was performed using DEGseq (Wang et al., 2010a). miRNAs with | log_2_ (fold change)| ≥ 1.0 between samples and *p*-value ≤0.05 were considered to be DEG miRNAs.

To validate the sRNA sequencing data, qRT-PCR was carried out. Total RNA containing sRNAs was first polyadenylated and then reverse-transcribed to cDNA using the Mir-X^TM^ miRNA First-Strand Synthesis Kit (Clontech, CA, United States). Real-time PCR was performed on a LightCycler^®^ 96 System (Roche) with an SYBR Premix Ex Taq^TM^ kit (Takara, Japan). All reactions were performed in triplicate. The forward primers were designed based on the mature miRNA sequences, and the universal reverse primer was supplied in the Mir-X^TM^ miRNA First-Strand Synthesis Kit. The primer sequences are listed in Supplementary Table 1. The *U6* gene was used as an internal control for the normalization of qRT-PCR data. The expression levels of the miRNAs at 0 h were set as 1.0, and relative expression at other time points was calculated using the 2^–ΔΔ^ ^CT^ method (Livak and Schmittgen, 2001).

### Prediction and Validation of the Target Genes of miRNAs

The potential target genes of cotton miRNAs were predicted using TargetFinder software (alignment score ≤4) (Kielbasa et al., 2010). To validate the predicted target genes, RNA ligase-mediated 5′- rapid amplification of cDNA ends (RLM-5′ RACE) was performed using a GeneRacer kit (Invitrogen). Total RNA from the cotton internode was directly ligated to the RNA oligo adapter. First-strand cDNA was reverse-transcribed using oligo dT primers and Superscript II reverse transcriptase (Invitrogen). Nested PCR was performed using 5′-adaptor primers and 3′ gene-specific primers (Supplementary Table 2). After amplification, PCR products with expected sizes were gel-purified using a QIAquick^®^ Gel Extraction Kit (QIAGEN, Valencia, CA, United States) and cloned into the pMD19-T vector (Takara, Japan). Following transformation, positive *Escherichia coli* DH5α clones identified using colony PCR were used for isolation of plasmids, which were subjected to Sanger sequencing (Li et al., 2010).

## Results

### Overview of the sRNA Library Sequencing Data

**FIGURE 1:**
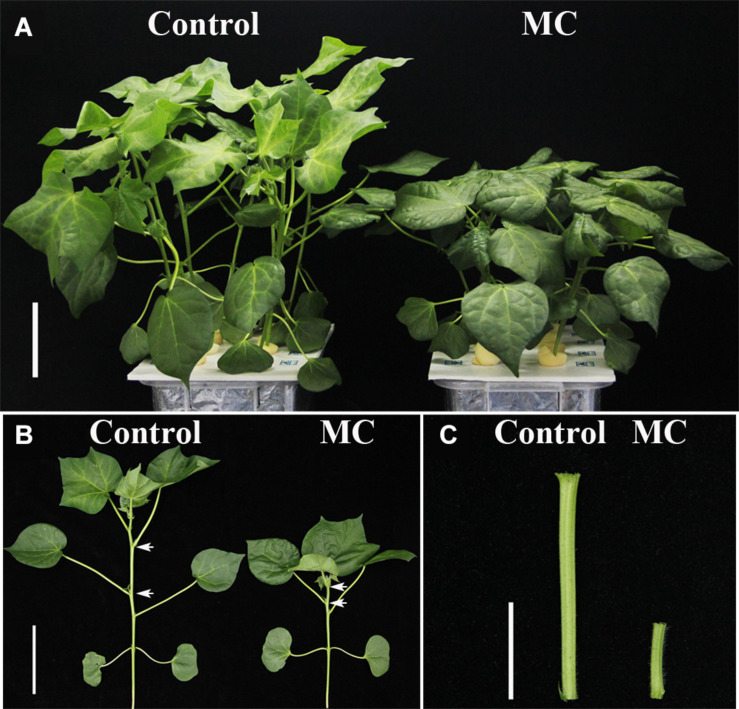
Morphological phenotypes of MC-induced growth inhibition of cotton seedlings. **(A,B)** Control and MC-treated seedlings at 10 d post treatment. The white arrows indicate the second internode. Bar = 10 cm. **(C)** The second internode of control and MC-treated seedling at 10 d post treatment. Bar = 3 cm.

**FIGURE 2:**
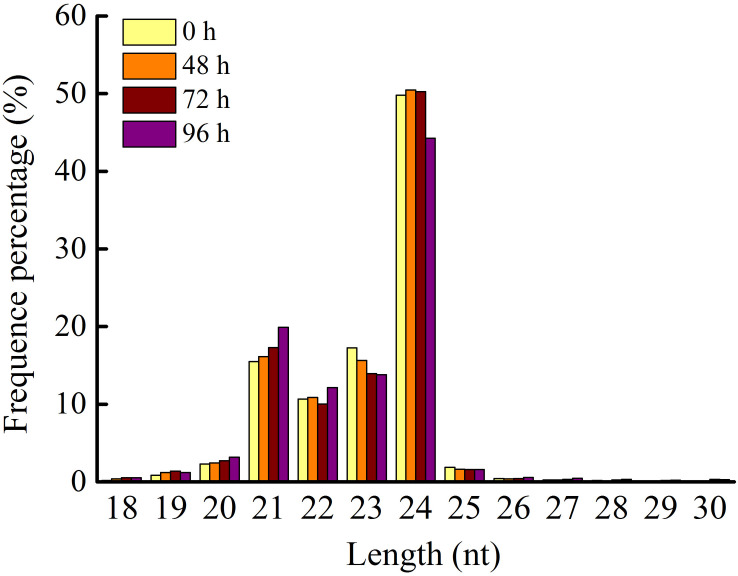
Length distribution of sRNA in the four small RNA libraries. 0, 48, 72, and 96 h represent four time points post MC treatment.

Application of 80 mg/L MC significantly reduced the internode length and plant height of cotton seedlings (Figure 1), corroborating the result of our previous study (Wang L. et al., 2020). Previously, we found that the transcriptional levels of genes related to GA biosynthesis and signaling and cell expansion were inhibited by MC after 48 h of treatment owing to the time-dependent translocation and accumulation of MC from the leaf to the internode (Wang L. et al., 2020). Therefore, the second elongating internodes were collected at four time points (0, 48, 72, and 96 h) post MC treatment for sRNA library preparation and MC-responsive miRNA identification. After discarding low-quality reads and contaminated sequences and adaptor trimming, a total of 98,906,991 clean reads representing 52,952,581 unique reads, ranging from 18 to 30 nt, were retained for subsequent analysis (Table 1). The size distributions of the four sRNA libraries showed similar patterns (Figure 2). The 24-nt reads were the most abundant class (about 50%), followed by 21-nt reads (17%) (Figure 2), in agreement with previous reports on cotton and other plant species (An et al., 2015; Li et al., 2019; Zhao et al., 2020). On the average, 84.18% of the reads were successfully aligned to the AD genome of *G*. *hirsutum*. The reads mapping to tRNAs, rRNAs, snoRNAs, and snRNAs accounted for 0.00–2.35% of the total reads (Table 1). The sequencing data and mapping statistics reflect the high-quality sRNA libraries obtained in this study.

**TABLE 1:** Read statistics of four cotton internode small RNA libraries.

Type	Raw reads	Clean reads	Mapped reads	rRNA	snRNA	snoRNA	tRNA	Unique reads	Unique mapped reads
0 h	25,516,715	24,556,182 (100%)	20,776,551 (84.61%)	309,986 (1.21%)	13 (0.00%)	338 (0.00%)	13,392 (0.05%)	14,262,096 (100%)	11,235,293 (78.78%)
48 h	23,951,610	23,159,571 (100%)	19,102,915 (82.48%)	413,155 (1.72%)	17 (0.00%)	269 (0.00%)	12,564 (0.05%)	12,760,520 (100%)	9,691,613 (75.95%)
72 h	26,285,348	25,272,630 (100%)	21,403,498 (84.69%)	386,318 (1.47%)	18 (0.00%)	348 (0.00%)	13,802 (0.05%)	14,248,382 (100%)	11,300,636 (79.31%)
96 h	27,129,484	25,918,608 (100%)	22,014,595 (84.94%)	636,338 (2.35%)	22 (0.00%)	407 (0.00%)	15,948 (0.06%)	11,681,583 (100%)	9,138,581 (78.23%)
Total	102,883,157	98,906,991 (100%)	83,297,559	1,745,797	70	1,362	55,706	52,952,581	41,366,123

*Data in parentheses are the percentages.*

### Identification of Known miRNAs in the Elongating Internode

To identify known cotton miRNAs in the sRNA libraries, we downloaded the mature miRNA sequences from all plant species deposited in miRBase 22.1 and combined them to obtain all known plant miRNA sequences. The unique reads from the four cotton sRNA libraries were subjected to a BLASTN search against all known plant miRNA sequences. The sRNAs that matched the known plant miRNA sequences with fewer than two mismatches were annotated as known cotton miRNAs. In total, 508 known miRNAs belonging to 197 families were identified in the four libraries (Supplementary Table 3). Among them, 21 highly conserved miRNA families were identified, and they included: miR156/157, miR159, miR160, miR162, miR164, miR165/166, miR167, miR168, miR169, miR170/171, miR172, miR319, miR390, miR393, miR394, miR395, miR396, miR397, miR398, miR399, and miR408. Currently, there are 378 mature miRNAs belonging to 217 families deposited in miRBase (22.1) for the genus *Gossypium* (*Gossypium raimondii*, *Gossypium hirsutum*, *Gossypium arboreum*, and *Gossypium herbaceum*). In this study, 309 cotton miRNAs belonging to 177 families (81.7%) were identified, indicating a good sequencing coverage of the four sRNA libraries.

The expression of known miRNAs was found to vary significantly. The highly abundant miRNAs were conserved miRNAs such as miR159, followed by miR166, miR164, miR319, miR168, miR171, miR156, miR398, and miR396. Several conserved miRNAs such as miR397, miR399, and miR172 showed relatively low expression levels. Most of the less conserved known miRNAs exhibited low expression levels; however, the abundance of miR535, miR2949, miR482, miR2947, miR3476, and miR7505 was high at different time points.

### Identification of Novel miRNAs in the Elongating Internode

Excluding the identified known miRNAs, the remaining sRNA sequences were used to predict potential novel miRNAs using the miRDeep2 software. We further manually checked the hairpin structure of the predicted novel miRNAs using MFOLD software (Zuker, 2003), and only those with the typical stem-loop structure were retained. Five novel miRNAs were identified in this study and were designated as ghr-miRn1 to ghr-miRn5 (Supplementary Table 4 and Supplementary File 1), and the corresponding miRNA^∗^ sequences were also detected. The lengths of these novel miRNAs and miRNAs^∗^ varied from 20 to 24 nt. Compared with conserved miRNAs, the abundance of most novel miRNAs was relatively low (Table 2). In addition, three novel miRNA members, which belong to known miRNA families (miR390, miR3627, and miR2275), were identified in this study (Supplementary Table 4).

**TABLE 2:** Expression levels of novel miRNAs identified from cotton internode in response to MC.

miRNA_id	miRNA expression (TPM)				Sequence
	0 h	48 h	72 h	96 h	
**Novel miRNAs**					
ghr-miRn1-5p	1.10	7.64	9.58	0.12	TTGTCCACGCGCGACACGCAC
ghr-miRn1-3p	1.55	0.99	0.63	0.96	GTGTTTCGCGCGTGGACGAC
ghr-miRn2-5p	1.26	1.64	4.43	1.16	TGTCGCAGGAGCGATGGCACTG
ghr-miRn2-3p	0.61	5.74	8.51	0.77	GTGCCATCGGCCTGCGACAAG
ghr-miRn3-5p	33.88	13.77	38.34	40.51	ACAGGTGGTGGATCAAATATGAGT
ghr-miRn3-3p	2.00	0.47	0.24	0.46	TCATATTTGTTCCACCCGCCTGTG
ghr-miRn4-5p	2.73	2.76	1.50	3.94	AGTCTCCTTCAAACGCTTCCAG
ghr-miRn4-3p	1.87	12.18	5.14	11.65	GGAAGGTTTGGAGGAGAGTGA
ghr-miRn5-5p	6.96	3.76	7.44	0.66	CAAGGCTTTGGGATACAAG
ghr-miRn5-3p	11.77	1.38	10.92	13.35	TGTATTTCAAAGCCTTGGTT
**Novel conserved miRNAs**					
cotton-miR3627-5p	0.29	0.78	2.53	0.42	TTGTCGCAGGAGCGATGGCACT
cotton-miR3627-3p	2.12	8.38	6.09	0.73	GTGCCTTCGGCCTGCGACAAG
cotton-miR2275-5p	0.94	0.52	1.74	0.15	TTTTTCACAAATATCACAATA
cotton-miR2275-3p	54.41	41.84	58.56	15.05	TTGTGATATTAGTGAAAAACA
cotton-miR390-5p	18.98	18.05	33.44	38.27	GAAACTCAGGATAGATAGCGC

### Identification of DEG miRNAs in Elongating Internode After MC Application

To identify MC-responsive miRNAs in cotton internodes, differential expression analysis of the miRNAs was performed between control (0 h) and MC treatment (48, 72, and 96 hpt). Based on the strict criteria (| log_2_ (fold change)| ≥ 1, *p*-value ≤ 0.05, and TPM ≥ 5 in at least one sample), a total of 104 DEG miRNAs were identified (Supplementary Table 5). Sixty-three DEG miRNAs (61%) were highly conserved miRNAs, and 20 were cotton-specific miRNAs, which have been identified only in cotton. Compared with the control (0 h), 60 (24 upregulated and 36 downregulated), 65 (34 upregulated and 31 downregulated), and 77 (29 upregulated and 48 downregulated) DEG miRNAs were identified at 48, 72, and 96 hpt, respectively. The number of DEG miRNAs increased with time post treatment.

**FIGURE 3:**
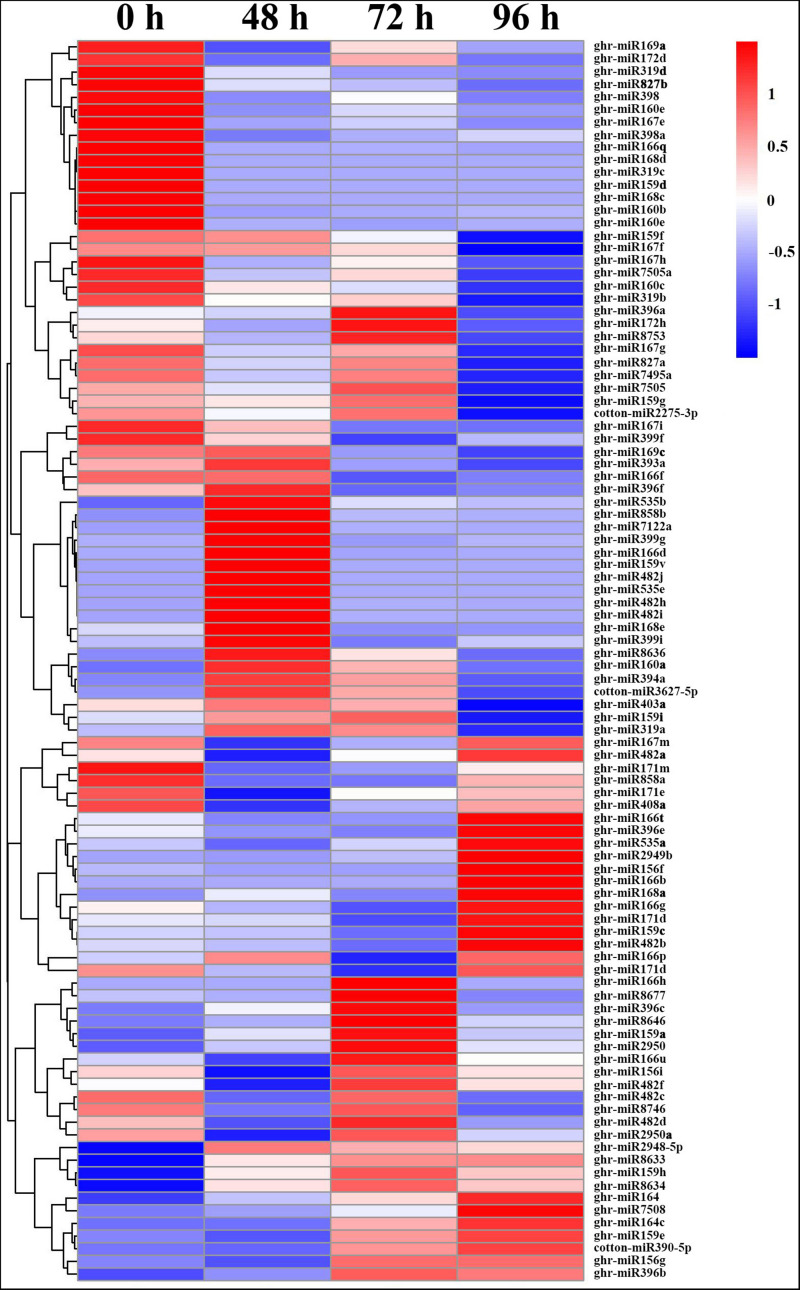
Heat map of differentially expressed miRNAs in cotton internode between MC treatment and control. Data was presented as miRNA abundances (TPM). Blue indicates lower expression, and red indicates higher expression. 0 h: Control; 48, 72 and 96: 48, 72, and 96 h post MC treatment.

Generally, more downregulated miRNAs were detected than upregulated miRNAs, except at 72 hpt. All members of miR167 and miR827, and most members of miR160, miR168, miR169, miR171, miR172, and miR319 were downregulated by MC treatment (Figure 3 and Supplementary Table 5). Among them, 14 miRNAs were continuously repressed by MC treatment, including miR159d, miR160 (b, e), miR166 (e, q), miR167e, miR168 (c, d), miR171m, miR319 (c, d), miR398, miR398a, and miR827b. In particular, miR168c, miR319c, and miR159d showed more than nine-fold downregulation. In contrast, 12 miRNAs were continuously induced by MC, including miR159 (h, v), miR482 (h, i, j), miR535 (b, e), miR858b, miR2948-5p, miR7122a, miR8633, and miR8634. Most of them exhibited more than a twofold upregulation. For novel DEG miRNAs, only ghr-miRn4 showed a continuous upregulation. Most DEG miRNAs were altered in response to MC at early or late post treatment time (Figure 3 and Supplementary Table 5). For example, miR858a showed more than twofold downregulation at 48 and 72 hpt; miR164 members showed significant upregulation at 72 and 96 hpt, with miR164c being upregulated by more than sixfold; some cotton-specific miRNAs such as miR2275, miR7495a, miR7505, miR7505a, and miR8753 were significantly downregulated only at 96 hpt. These results suggest the importance of miRNA-mediated post-transcriptional regulation in MC-induced inhibition of internode elongation.

**FIGURE 4:**
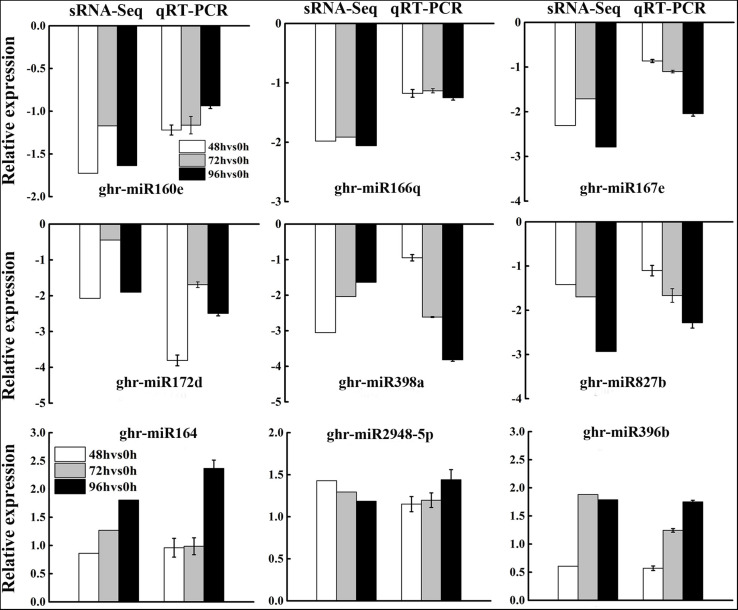
Validation of differentially expressed miRNAs from sRNA-Seq analysis by qRT-PCR. Data from sRNA-Seq was presented as the log_2_ fold change values at 48, 72, and 96 h post MC treatment compared to control (0 h), while that from qRT-PCR was presented as –delt delt Ct. Each column represents mean ± SD. Error bars represent the SD of three biological and three technical replicates.

To validate the expression patterns of DEG miRNAs obtained by sRNA-Seq, three upregulated and six downregulated miRNAs were selected for qRT-PCR. In general, the expression patterns of most DEG miRNAs obtained from qRT-PCR were in accordance with those obtained by deep sequencing (Figure 4), although the fold change of expression detected by qRT-PCR was not completely consistent with the sRNA-Seq results owing to the difference in sensitivity and specificity between the two techniques. The results showed that the expression profiles of miRNAs using deep sequencing were reliable for further analyses.

### Target Prediction of Known and Novel miRNAs

MicroRNAs regulate many biological processes by regulating the expression of target genes. To better understand the role of miRNAs in MC-induced inhibition of internode elongation, we performed target prediction of miRNAs using the TargetFinder program (Kielbasa et al., 2010), and targets with scores less than four were considered putative cotton miRNA targets. In total, 1,638 targets for known miRNAs and 141 targets for novel miRNAs were predicted (Supplementary Table 6). Many targets of the conserved miRNAs were TFs, including SPL (miR156), MYB (miR159), NAC (miR164), homeobox-leucine zipper protein ATHB (miR166), nuclear transcription factor Y subunit A (miR169), TCP (miR319), and GRF (miR396). Some targets are involved in plant hormone signaling pathways. For example, auxin response factor (ARF) (miR160 and miR167), transport inhibitor response 1 (TIR1) (miR393), and auxin signaling F-box 2-like protein (miR393) are involved in the auxin signaling pathway; scarecrow-like protein (SCL) (miR171) is related to the GA signaling pathway. In addition to the previously identified conserved targets, many new targets have also been identified for known miRNAs. These novel targets include cytokinin dehydrogenase 1-like (miR159), indole-3-acetaldehyde oxidase-like (miR7505), and GA 3 oxidase 1 (miR7508), which are involved in plant hormone metabolism, indicating that these miRNAs could mediate plant hormone metabolism and signaling.

**FIGURE 5:**
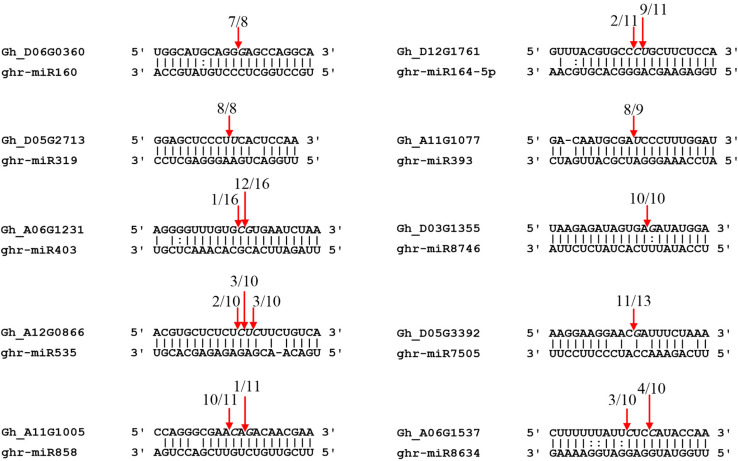
Validation of miRNA targets by RLM-5′ RACE. Red arrows indicate the cleavage sites detected by RLM-5′ RACE.

RLM-5′ RACE is a widely used technique for verifying miRNA-mediated cleavage of target genes. Thirteen predicted targets were selected to perform the RLM-5′ RACE assay, and 10 genes were confirmed as miRNA targets. Among them, four conserved targets for miR160 (Gh_D06G0360, ARF17), miR164 (Gh_D12G1761, NAC100), miR319 (Gh_D05G2713, GAMYB), and miR393 (Gh_A11G1077, TIR1) were identified (Figure 5). Six novel targets were validated in cotton for the first time: AGO2 (Gh_A06G1231), SPL7 (Gh_A12G0866), pentatricopeptide repeat-containing protein (PPR, Gh_D05G3392), MYB4 (Gh_A11G1005), cysteine proteinase inhibitor (CYSb, Gh_A06G1537), and putative disease resistance protein RGA1 (Gh_D03G1355), which are targeted by miR403, miR535, miR7505, miR858, miR8634, and miR8746, respectively. It is worth noting that miR8746, miR7505, and miR8634 are cotton-specific miRNAs. All 10 target genes had specific cleavage sites located in the middle of the complementary sequences between miRNAs and their target sites.

## Discussion

Mepiquat chloride is a widely used growth regulator for improving cotton architecture, producing compact plants by inhibiting internode elongation and reducing leaf area. Recently, we found that MC significantly altered the expression of a large number of genes related to cell cycle, cell wall structure, hormone metabolism, signal transduction, and secondary metabolism, and TFs (Wang L. et al., 2020). miRNA is a kind of important post-transcriptional regulator of gene expression. The aim of the present study was to reveal the roles of miRNAs in MC-induced growth inhibition in cotton.

In the present study, a total of 508 known miRNAs from 197 families and five novel miRNAs were identified from the elongating internode of cotton seedlings (Supplementary Tables 3,4). This number is much higher than that previously reported for cotton (*G. hirsutum*) stem apexes (An et al., 2015). Among the conserved miRNAs, miR159 was the most abundant in the elongating internode, followed by miR166, miR164, miR319, miR168, and miR171 (Supplementary Table 3). High expression of miR159, miR166, and miR171 was also found in cotton stem apexes (An et al., 2015). In addition, other miRNAs such as miR535, miR2949, miR482, and miR2947 were also highly expressed in the elongating internode (Supplementary Table 3), and similar results were observed in stem apexes of cotton, except for miR2947, which was not found in the previous study (An et al., 2015). The high miRNA diversity in internodes implies that a complex mechanism is involved in cotton growth and development.

**FIGURE 6:**
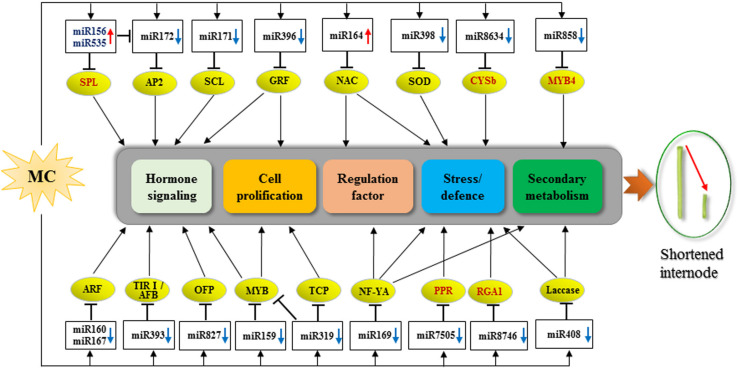
Proposed model for the roles of miRNA-targets in MC-induced inhibition of internode elongation. Up and down arrows behind the miRNA names represent up- and down-regulation by MC, respectively. The targets in red color are novel targets validated in this study. CYSb, cysteine proteinase inhibitor; PPR, pentatricopeptide repeat-containing protein; RGA1, resistance gene analog 1.

A total of 104 DEG miRNAs were found to be responsive to MC (Figure 3 and Supplementary Table 5). Sixty-three DEG miRNAs (61%) were highly conserved. miRNAs regulate many biological processes through mRNA cleavage or translational repression. Identification of miRNA targets is crucial for understanding miRNA-mediated processes. In this study, 1,638 targets for known miRNAs and 141 targets for novel miRNAs were predicted based on bioinformatics analysis (Supplementary Table 6). Many conserved targets were TFs such as GRF, TCP, MYB, SPL, ARF, NAC domain, and SCL (Supplementary Table 6). Previously, we identified 497 differentially expressed TFs in response to MC (Wang L. et al., 2020); among them, 36 TFs were found to be targeted by miRNAs in this study (Supplementary Table 6). In addition to the known targets, many novel targets of known miRNAs and novel miRNAs were predicted (Supplementary Table 6). Given the false-positive rate of computationally predicted targets, experimental confirmation of these targets is necessary. Degradome sequencing provides a high-throughput strategy for the global validation of miRNA targets (Song et al., 2017). An et al. (2015) validated the interaction between miR172 and AP2; miR160 and ARF; and miR159, miR319, and miR858 and MYB based on the released cotton degradome sequencing data. In this study, we confirmed 10 miRNA target genes using RLM-5′ RACE. Among them, six genes were verified as novel targets of six miRNAs for the first time. It is worth noting that Gh_D05G3392 (PPR), Gh_A06G1537 (CYSb), and Gh_D03G1355 (RGA1) were targeted by cotton-specific miR7505b, miR8634, and miR8764, respectively (Figure 5). Based on the analysis of miRNA response to MC, we present a model to reveal the regulatory network between miRNAs and their targets involved in MC-induced inhibition of internode elongation (Figure 6).

Our previous study revealed that MC repressed both cell division and cell elongation by downregulating the expression of many genes related to cell cycle and cell wall architecture, leading to shortened internodes (Wang L. et al., 2020). Several conserved miRNAs such as miR396, miR319, and miR159 have been reported to control cell proliferation (Rodriguez et al., 2016). miR396 negatively regulates cell proliferation by targeting GRF TFs, which are usually strongly expressed in actively growing tissues (Rodriguez et al., 2016). Overexpression of miR396 in Arabidopsis and rice suppressed the expression of GRFs and cell cycle-related genes, leading to dwarfism (Rodriguez et al., 2010; Tang et al., 2018). Our result showed that the expression of three miR396 members was significantly induced by MC at 72 and 96 hpt (Figure 3 and Supplementary Table 5), corroborating the result of our previous study, which revealed that 12 GRFs showed reduced expression in response to MC (Wang L. et al., 2020). High expression of miR396 was also reported in the stem apexes of cotton dwarf mutant and shoots of apple dwarf mutant (An et al., 2015; Song et al., 2017). miR396 was found to be negatively regulated by GA during cell proliferation (Lu et al., 2020). Our previous study showed that MC significantly reduced GA levels in cotton internodes (Wang L. et al., 2020). Thus, the low GA concentration caused by MC may promote the expression of miR396, resulting in suppressed expression of GRFs and cell cycle-related genes, thus leading to inhibited internode elongation in cotton, as in rice (Lu et al., 2020).

miR319 has been demonstrated to promote cell proliferation during leaf and petal development (Nag et al., 2009; Liu et al., 2020); however, the effect of miR319 on stem elongation varies with plant species. Overexpression of sha-miR319d in tomato reduced plant height (Shi et al., 2019), whereas overexpression of osa-miR319b in switchgrass promoted stem elongation and increased plant height (Liu et al., 2020). Knockdown of miR319 using artificial miRNA target mimics (MIM) completely suppressed stem elongation in Arabidopsis (Todesco et al., 2010). In this study, all miR319 members showed significant downregulation at 96 hpt, with ghr-miR319c being downregulated by more than 10-fold (Figure 3 and Supplementary Table 5). Our results suggest a positive role of miR319 in cotton internode elongation. miR159, similar in sequence to miR319, positively regulates organ size by promoting cell division (Rodriguez et al., 2016). In rice, suppressing miR159 through short tandem target mimic (STTM) resulted in short internodes and small leaves owing to the disruption of cell division, which is related to the reduced expression of genes associated with cell cycle and hormone homeostasis (Zhao et al., 2017). The Arabidopsis *mir159ab* double mutant showed a dwarf phenotype (Allen et al., 2007). The expression of four miR159 members was significantly downregulated in response to MC, and the expression of ghr-miR159d was downregulated by more than ninefold (Figure 3 and Supplementary Table 5). In addition, one MYB TF DIVARICATA (DIV) (Gh_D04G0641), a target of miR159, was upregulated by MC in cotton internodes in our previous study (Wang L. et al., 2020). DIV was first reported to modulate the growth of ventral and lateral petals (Galego and Almeida, 2002). Recently, DIV has been suggested to regulate cell proliferation during stamen development in the flowers of *Plantago* (Reardon et al., 2014) and control cell expansion in the fruit pericarp of *Solanum lycopersicum* by interacting with other proteins (Machemer et al., 2011). The increased expression of DIV may contribute to the inhibition of cell division and elongation of internodes under MC treatment. These results indicate that decreased expression of miR159 members is associated with MC-induced inhibition of internode elongation, which is similar to the findings in Arabidopsis (Todesco et al., 2010) and rice (Zhao et al., 2017).

miR156 negatively regulates plant height by repressing the expression of SPLs. The dwarf phenotype has been observed in transgenic Arabidopsis, rice, maize, alfalfa, and switchgrass overexpressing miR156 (Schwab et al., 2005; Chuck et al., 2007; Fu et al., 2012; Gao et al., 2016; Dai et al., 2018). Thus, as expected, the expression of ghr-miR156f and ghr-miR156g was induced by MC in cotton internodes (Figure 3 and Supplementary Table 5). miR535 shows extremely high sequence identity to miR156 sequence, and six SPLs (OsSPL4/7/11/12/16/19) were co-targeted by miR156/miR535 in rice (Liang et al., 2013; Sun et al., 2019). Overexpression of either miR535 (Sun et al., 2019) or miR156 (Xie et al., 2006) in rice shortened the internode length and thus reduced plant height by down-regulating OsSPL7. Similar to miR156, the expression of miR535 was also significantly induced by MC (Figure 3 and Supplementary Table 5), suggesting their overlapping functions in inhibiting cotton internode elongation. Our previous study demonstrated that 12 SPLs, including four SPL7 TFs, which are homologous to OsSPL7, showed significant downregulation in response to MC (Wang L. et al., 2020); this correlates well with the increased levels of miR156 and miR535. Moreover, Gh_A12G0866 (GhSPL7) was confirmed to be cleaved by miR535 in cotton using the RLM-5′ RACE assay (Figure 5).

miR172 has been reported to act downstream of miR156 (Wu et al., 2009; Jung et al., 2011). These two miRNAs often show inverse expression patterns during plant growth and development in many plant species (Chuck et al., 2007; Wu et al., 2009; Wang et al., 2011; Bhogale et al., 2014). miR156 represses the transcription of miR172 via SPL genes in Arabidopsis, *Populus* × *canadensis*, and potato (Wu et al., 2009; Wang et al., 2011; Bhogale et al., 2014). Thus, the reduced expression of ghr-miR172d and ghr-miR172h might be ascribed to the increased expression of miR156 under MC treatment (Figure 3 and Supplementary Table 5). In rice, silencing of miR172 by STTM led to severe defects in internode elongation and resulted in dwarfism (Zhang et al., 2017). Therefore, miR156 may negatively regulate internode elongation by down-regulating SPLs and miR172 in cotton under MC treatment.

miR160 mediates auxin signaling by restraining certain ARFs and further regulating plant growth (Liu et al., 2010; Zhang et al., 2017). Silencing of miR160 by STTM in Arabidopsis and rice resulted in dwarf stature (Liu et al., 2010; Zhang et al., 2017). The expression of three miR160 members was significantly downregulated by MC, and ghr-miR160b exhibited more than fourfold downregulation (Figure 3 and Supplementary Table 5). GhARF18 (Gh_A13G2013), a target of miR160, was found to be significantly upregulated by MC in our previous study (Wang L. et al., 2020), which correlates well with the repression of miR160. In polyploid rapeseed, ARF18 was reported to inhibit downstream auxin genes during silique development (Liu et al., 2015). Rice transgenic plants, expressing an osa-miR160-resistant version of OsARF18, exhibited dwarf stature because of the disruption of auxin signaling (Huang et al., 2016). In addition, the transcripts of GhARF17 (Gh_D06G0360) were cleaved at the miR160 complementary site in the cotton internode (Figure 5). These results suggest that the miR160-ARF module plays an important role in the MC-induced inhibition of internode elongation by modifying auxin signaling.

miR164 has been shown to participate in development and stress defense in plants by targeting different NAC-domain genes, including uncharacterized CUP-SHAPED COTYLEDON (*CUC*). In rice, knocking out *OsCUC1*, a target of osa-miR164c, leads to a dwarf plant architecture (Wang J. et al., 2020). Overexpression of miR164b or downregulation of its target, OsNAC2, led to reduced plant height in rice (Jiang et al., 2018). In the present study, all miR164 members were induced by MC treatment, and ghr-miR164c was upregulated by more than sixfold at 72 and 96 hpt (Figure 3 and Supplementary Table 5). Increased expression of miR164 was also found in the stem apexes of cotton dwarf mutants (An et al., 2015). In accordance with the upregulation of miR164, eight NAC TFs, including CUC2 (a target of miR164, Gh_D01G0448), CUC3, and NAC2, were found to be downregulated by MC in cotton internodes in our previous study (Wang L. et al., 2020). In addition, GhNAC100 (Gh_D12G1761) was found to be cleaved by miR164 in cotton internodes (Figure 5). A recent study showed that ghr-miR164 could improve cotton plant resistance to *Verticillium dahlia* by downregulating GhNAC100 (Gh_A11G0290) (Hu et al., 2020). These results suggest that the upregulation of miR164 by MC may not only result in the inhibition of internode elongation but also improve the resistance of cotton plants to the stress caused by MC.

miR171 controls shoot development by targeting SCL6 (Wang et al., 2010b). The expression of all miR171 members was downregulated by MC (Figure 3 and Supplementary Table 5), which correlates well with our previous study, which showed that two SCL6 genes (Gh_A12G0855 and Gh_D12G0935), targets of miR171, were significantly upregulated by MC (Wang L. et al., 2020). Decreased expression of miR171 was also found in the shoots of apple dwarf mutants (Song et al., 2017). Silencing of miR171 in rice caused semi-dwarf stature (Zhang et al., 2017). In contrast, overexpression of miR171 in Arabidopsis and rice or triple *scl6* mutant Arabidopsis resulted in increased plant height (Wang et al., 2010b; Fan et al., 2015). Therefore, miR171 is a positive regulator of plant height.

miR398 has been reported to function in various stress responses by repressing Cu/Zn superoxide dismutases (Sunkar et al., 2006). Little is known about the role of miR398 in plant growth and development. Recently, a positive role of miR398 in plant height has been identified based on the dwarf phenotype of STTM398 lines and increased height of miR398-overexpressing lines in rice (Zhang et al., 2017). In the present study, all miR398 members were repressed by MC (Figure 3 and Supplementary Table 5), suggesting that miR398 is not only related to stress response but also might be associated with cotton internode elongation.

Besides these conserved DEG miRNAs, miR827, and miR858 were also strongly responsive to MC (Figure 3 and Supplementary Table 5). Previous studies have reported the roles of miR827 in stress response and anther and fruit development (Liu et al., 2015; Nie et al., 2018; Fileccia et al., 2019). In this study, two miR827 members were significantly downregulated in response to MC (Figure 3 and Supplementary Table 5), and miR827 was predicted to target the transcription repressor OVATE family protein (OFP1, Gh_D10G1150) (Supplementary Table 6). Overexpression of OFPs in Arabidopsis, rice, and tomato has been reported to inhibit cell elongation and reduce plant height by suppressing GA biosynthesis (Wang et al., 2007; Schmitz et al., 2015; Zhou et al., 2019). Thus, we speculate that the suppressed expression of miR827 by MC may lead to the accumulation of OFP1 and further reduce GA content, resulting in the inhibition of internode elongation. miR858 has been reported to negatively regulate flavonoid biosynthesis by targeting different MYBs in different plant species (Jia et al., 2015; Sharma et al., 2016; Li et al., 2020). In Arabidopsis, miR858a positively regulates plant growth and lignin biosynthesis (Sharma et al., 2016). Overexpression of miR858a promoted vegetative growth; however, its knockdown using MIM reduced vegetative growth and the expression of lignin biosynthetic genes (Sharma et al., 2016). In this study, the expression of ghr-miR858a was downregulated by more than twofold by MC at 48 and 72 hpt (Figure 3 and Supplementary Table 5). Using the RLM-5′ RACE assay, MYB4 (Gh_A11G1005) was confirmed as a bona fide target of miR858 (Figure 5). Reduced expression of genes related to lignin biosynthesis was observed upon MC application in our previous study (Wang L. et al., 2020). Therefore, similar to miR858a in Arabidopsis, ghr-miR858a may be a positive regulator of internode elongation in cotton.

Several cotton-specific miRNAs, such as miR2948, miR7505, miR8633, miR8634, and miR8746, also showed altered expression in response to MC. A few of them have been reported to be related to stress response, anther development, or fiber development in cotton (Xie et al., 2015b; Peng et al., 2018; Zhang et al., 2018). Previously, we found that MC regulated the transcription levels of other hormone-related genes in a GA-dependent manner (Wang L. et al., 2020). Further studies are required to investigate whether these MC-responsive miRNAs are related to the reduced endogenous GA levels in cotton internodes.

In summary, a total of 508 known miRNAs belonging to 197 families and five novel miRNAs were identified from the elongating internode of cotton seedlings. Furthermore, 104 DEG miRNAs were identified as MC-responsive miRNAs. Most of the DEG miRNAs (61%) were highly conserved miRNAs. The expression of 14 known miRNAs was continuously suppressed, whereas 12 known miRNAs and one novel miRNA were continuously induced post MC treatment. Moreover, six novel target genes were verified using RLM-5′ RACE. This study deepens our understanding of the miRNA-mediated regulation network in cotton internode elongation and MC-mediated growth inhibition and could provide guidance for breeding new cotton varieties with better plant architecture. Future studies should focus on the detailed mechanisms of how these miRNAs are involved in the MC-mediated inhibition of internode elongation.

## Data Availability Statement

The datasets presented in this study can be found in online repositories. The names of the repository/repositories and accession number(s) can be found below: https://www.ncbi.nlm.nih.gov/, PRJNA647661.

## Author Contributions

Y-FL and LW conceived the idea and supervised the research. LW, YY, and XJ grew cotton seedlings and performed MC treatment, samples collection and RNA extractions. LW, YY, XJ, JY, and ZQ analyzed the sRNA library data sets. YY, XJ, MW, and MZ performed qRT-PCR analyses and miRNA target validation. LW and Y-FL wrote the manuscript. All authors read and approved the final manuscript.

## Conflict of Interest

The authors declare that the research was conducted in the absence of any commercial or financial relationships that could be construed as a potential conflict of interest.

## Footnotes

1 http://www.mirbase.org

2 http://rna.tbi.univie.ac.at/cgi-bin/RNAWebSuite/RNAfold.cgi
