# Supplementary material for: Profiling of MicroRNAs Involved in Mepiquat Chloride-Mediated Inhibition of Internode Elongation in Cotton (Gossypium hirsutum L.) Seedlings
Source: Front Plant Sci. 2021 Feb 24;12:643213. doi: 10.3389/fpls.2021.643213 (PMC7943613; doi:10.3389/fpls.2021.643213)

SUPPLEMENTARY FILE 1 Novel MIRNA structure and accumulation

1. Novel MIRNAs

ghr-MIRn1

Precursor

augacugcugaauuuuuguuguccacgcgcgacacgcacugcauuuguguuuuuucaaagcuuug
cauguGUGUUUCGCGCGUGGACGACguaaauuaacaagucauuau

Initial ΔG = -55.30

```
           10        20        30         40        50
---      gcug     u                c      -|   uuugu     u
   augacu    aauuu uguuguccacgcgcga acgcac ugca     guuuu \
   uacuga    uuaaa gcagcaggugcgcgcu ugugug acgu     cgaaa u
uau      acaa     u                u      u^   uu---     c
.       100        90        80        70           60
```

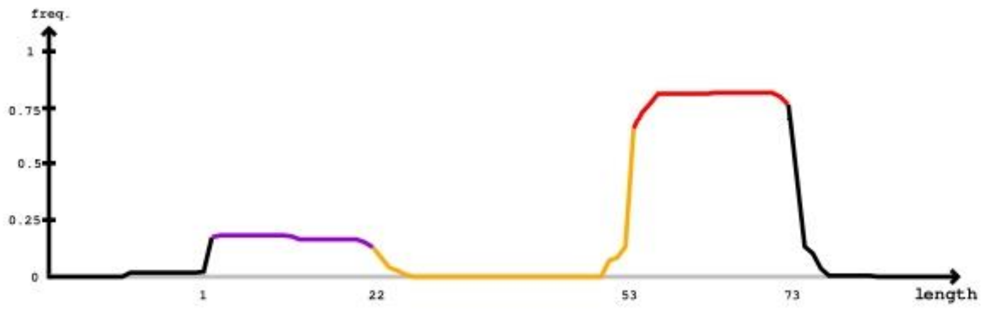

ghr-MIRn2

Precursor

acuggcgguuggggaaucuugucgcaggagcgauggcacugccuuuuguuuuuguuuuccuugu
auagcaacacagcacaGUGCCAUCGGCCUGCGACAAGguacccugca

Initial ΔG = -54.70

```
        10        20        30        40        50        60
acug| gguug    a             ag           cc  u    u    uuuc
    gc     gggg aucuugucgcagg  cgauggcacug  uu uguu uugu    \
    cg     uccc uggaacagcgucc  gcuaccgugac  ga acaa gaua    c
a---^ -----    a             g-           ac  c    c    uguu
   110            100         90        80        70
```

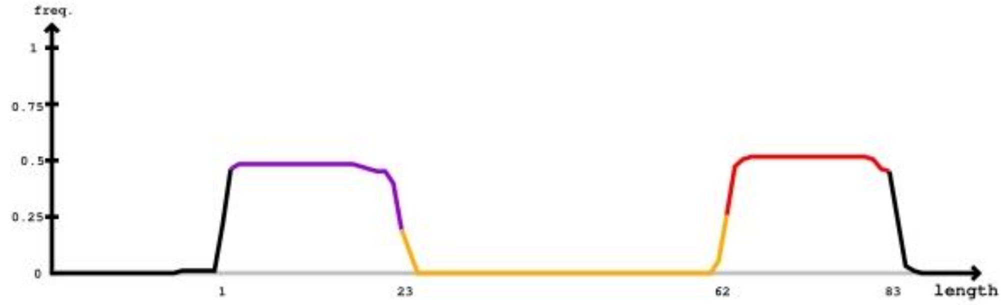

ghr-MIRn3

Precursor

ccaagguuguuuuucuguugacagguggugga ucaaauaugaguucuucaauguaaaaauUCAUA
UUUGUUCCACCCGCCUGUGGaugcaaaacaugacccugguaaacugcc

Initial ΔG = -44.50

```
                  10          20          30         40         50
---------   a   ug -     c     g       -|      u             c   ca
         cca ggu  u uuuu uguu acaggu ggugga caaauaugagu uu   a
         ggu cca  a aaaa guag uguccg ccaccu guuuauacuuaa aa   u
ccgucaaau   c   gu c     c     g       c^      u             a   ug
 110        100         90         80         70         60
```

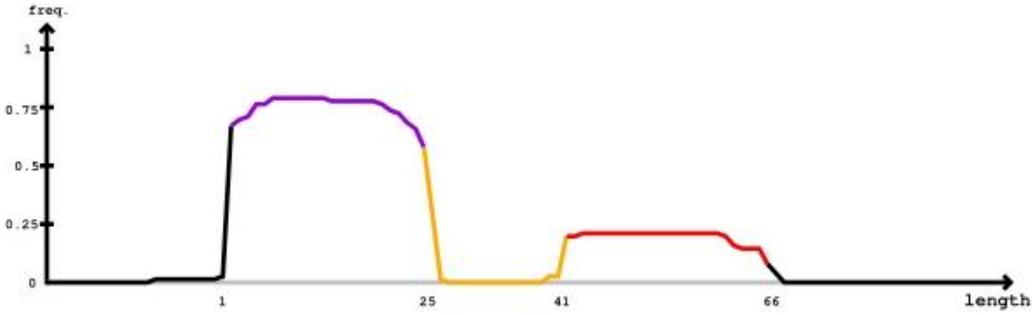

ghr-MIRn4

Precursor

gaauugguugcucAGUCUCCUUCAAACGCUUCCAGcaauauucuacacacuacccccccagcagca
cuguuggaagguuuggaggagagugaacaaaacaugccuugaug

Initial ΔG = -37.10

```
                10         20         30         40         50
gaauu-    ------| c   g            g         auauucuacacacu
      ggu       ug uca ucuccuucaaac cuuccagca              \
      ccg       ac agu agaggagguuug gaagguugu               a
guaguu    uacaaa^ a   g            -         cacgacgacccccc
       100        90         80         70         60
```

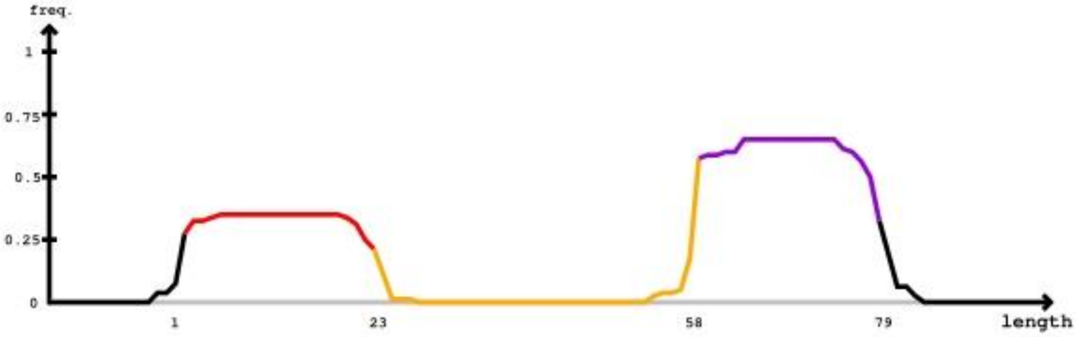

ghr-MIRn5

Precursor

uguauaacaccagcgauaaaCAAGGCUUUGGGAUACAAGcuuucugaugaauuacuuacauauua uguauguacaaagacugaguuuguauuucaaagccuugguuuu

Initial ΔG = -36.70

```
        10        20        30        40          50        60
uguauaacaccagcgauaaa          gg          --|  ga  aau   u     a
                    caaggcuuug  auacaagcuu  ucu  ug   uac uacau \
                    guuccgaaac  uauguuugag  aga  ac   aug augua u
uuuug---------------          uu          uc^  a-  ---   u     u
                     100        90        80            70
```

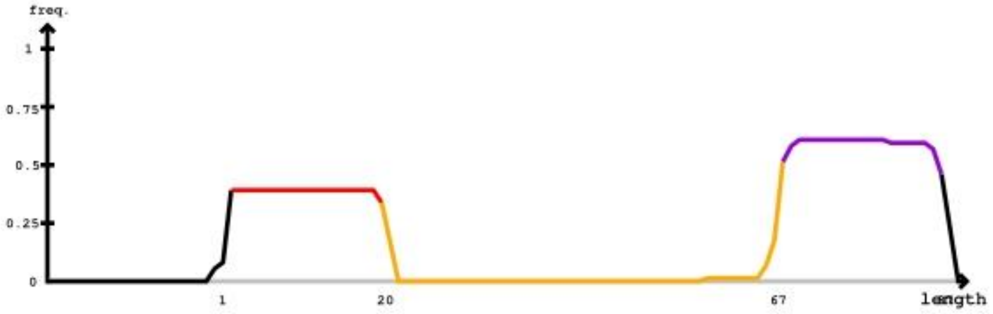

2.Novel conserved MIRNAs

cotton-MIR3627

Precursor

gggaaucUUGUCGCAGGAGCGAUGGCACUgccuuuuguuuuuguuuuccuuguauagcaacacag gacagugccuucggccugcgacaagguacccugcaacuucuuuuaa

Initial ΔG = -52.90

```
                       10        20        30         40
---------------|  a             ag   u       -   uu    u    uuuc
               ggg aucuugucgcagg  cga ggcacug ccu  uguu uugu    \
               ccc uggaacagcgucc  gcu ccgugac gga  acaa gaua    c
aauuuucuucaacgu^  a             g-   u       a   c-    c    uguu
 .       100        90        80         70         60        50
```

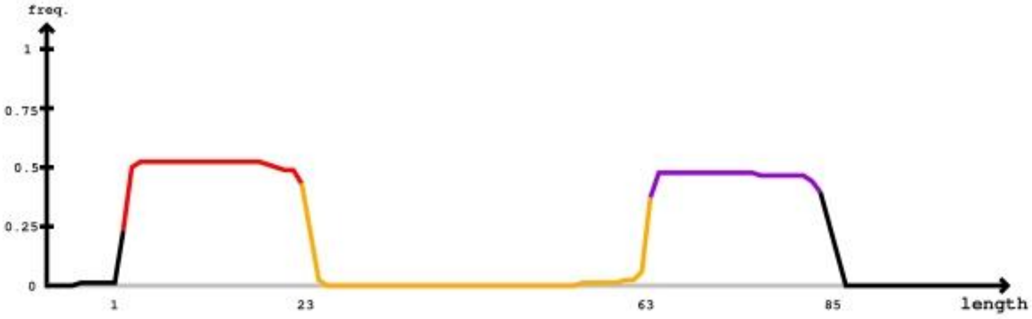

cotton-MIR2275

Precursor

aaucauuuucuuaccuucuuuuuuucacaaauaucacaauaaaacauggaugaaacuaucgauuaugauagaguucuauuaUUGUGAUAUUAGUGAAAAACAaaagguaca

Initial ΔG = -33.60

```
        10        20        30        40        50        60
aaucauuuucu|     cuu        a            aaac     ugaaa     ga
           uaccuu   uuuuucac aauaucacaaua    augga     cuauc  u
           auggaa   aaaaagug uuauaguguuau    uaucu     gauag  u
ac---------^     aac        a            ----     uga--     ua
 .                100        90        80              70
```

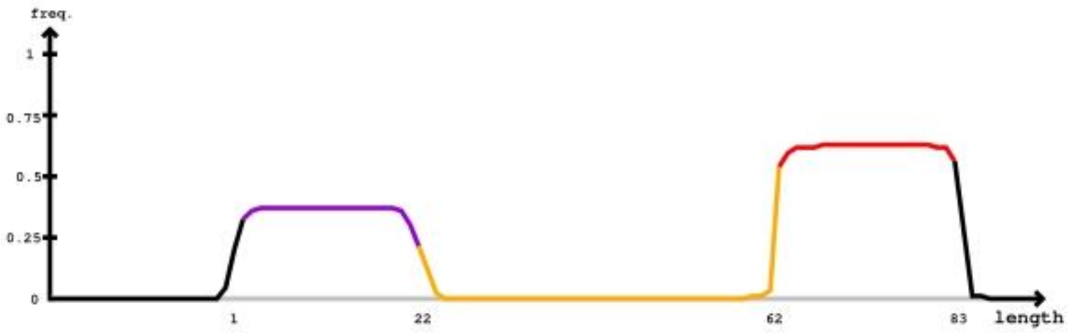

cotton-MIR390

Precursor

uauagcaggaagaacccauGAAACUCAGGAUAGAUAGCGCcauauauagguuuuuauuugagauggcgcuaucccuccugagcuuuacagguuaucccauacuugagguu

Initial ΔG = -41.90

```
              10        20        30        40        50
uauagca------|  ag    ca    a       ua           aua     u
             gga  aacc  ugaa cucagga  gauagcgccau   uaggu u
             ccu  uugg  auuu gaguccu  cuaucgcggua   guuua u
uuggaguucauac^  a-    ac    c       cc           ga-     u
.       100         90        80        70         60
```

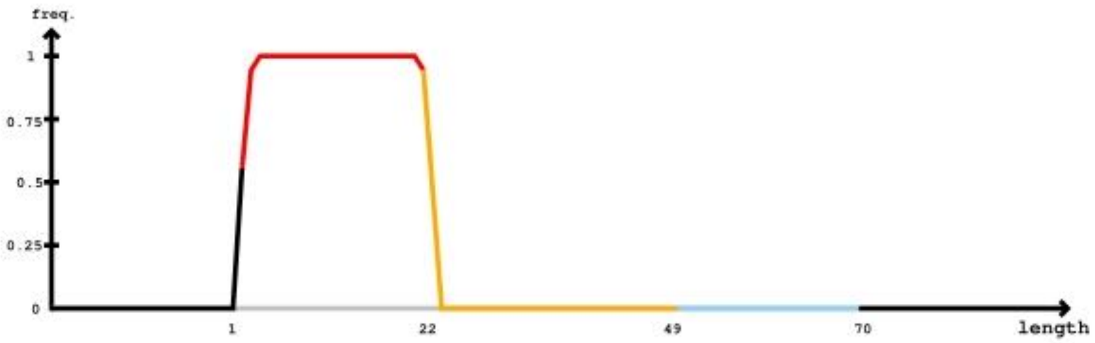

Supplement: Supplementary file 7 [file Data_Sheet_1.PDF]
